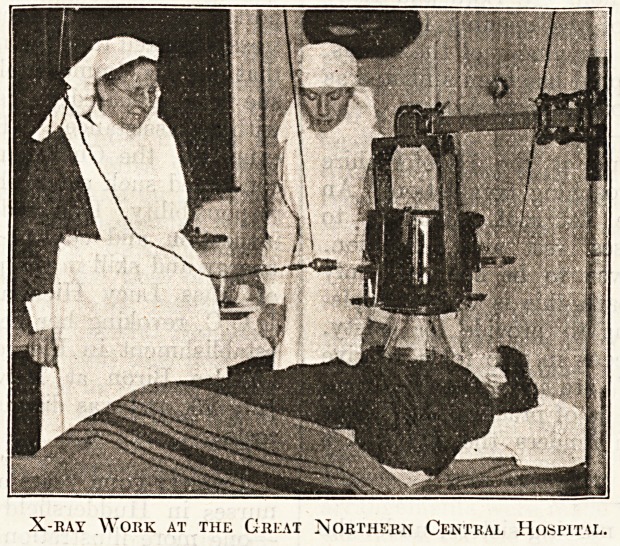# Qualifications and Study
*The first article appeared June 11.


**Published:** 1921-08-13

**Authors:** 


### WOMEN'S WORK IN THE X-RAY DEPARTMENT.

### II.—Qualifications and Study.*

By A FEMALE RADIOGRAPHER.

The qualifications to be acquired by the *x*-ray student may be divided into two sections—those that are absolutely necessary and those that are very desirable. The woman who wishes to take up *x*-ray as a career must begin from the very bottom rung of the ladder. The foundation of all our wonderful modern apparatus is electricity, therefore she must study electricity from its very rudiments, as a schoolboy is made to study it. She must study the human body from its very rudiments, as the V.A.D. has had to study it. Only she must leave the schoolboy and the V.A.D. far behind and mount to the level of the electrician and the doctor, if she can; and even this is saying nothing of the photographer.

It is somewhere between the schoolboy and the electrician, between the V.A.D. and the doctor, that we meet with the second section of radiographic knowledge. An *x*-ray department should have attached to it, for appeal whenever necessary, not only a trained radiologist, but also a skilled electrical engineer, and therefore it is not essential for the radiographer to reach either the level of the one in anatomy, or of the other in electricity; nevertheless, it is highly desirable.

Apart from the knowledge which is essentially that of the electrical engineer, and apart from the knowledge which is essentially that of the medical man, there is all the knowledge which is essentially that of the radiographer, who, alone of the three, is always on the spot, and to whom falls all the main work of the department. It is impossible in an article to dwell on this other than very lightly. The radiographer is entirely responsible for all the radiographs taken in the department, and must consequently be skilled in all the processes immediately attendant upon their production. The electrician has nothing to do with the *x*-ray tubes, which are sacred to the radiographer, and to be used only by her and such of her assistants as she trusts. The knowledge and proper use of the tubes is a science in itself. Tubes need to be discreetly chosen for the particular form of work on hand, and then wisely operated. They need studying, understanding, regulating, humouring, and nursing. More important than the particular make of any good tube is undoubtedly the skill of the operator.

X-ray Work at the Great Northern Central Hospital.

The *x*-ray plate also needs skilled treatment, and the radiographer must know how to make up her own developing, fixing, intensifying, and reducing solutions, and how to use them to the best advantage of the individual negatives. She must know all that there is to know about printing. Concerning therapy, the very least that she must be able to do is to operate in such a way as to carry out accurately specified treatments. Over and above that, there is no reason why she should not in time become as wise in the knowledge of *x*-ray therapy as the at present limited general knowledge on the subject permits; but she must never forget that she is dealing with a dangerous agent of cure, and that much of its responsibility is necessarily that of the radiologist.

Let us now briefly connect some of the necessary qualifications of the radiographer with those that are merely very desirable. The radiographer must know what will happen when she turns on certain switches, regulates certain handles, or makes certain connections; but it is very desirable for many obvious reasons that she should know *why* it happens. She must know when any of her apparatus is out of order; but it is a very good thing if she also knows how to put it right. She must be able to make up good developer for her dark room; but development only becomes an art when she knows the parts that are played by the different substances composing the whole, and only then can it be scientifically applied. She must be able to take good pictures and use the fluorescent screen; but it is also very desirable that she have a good eye for radio-diagnosis and a clear method of reporting her observations. In fact, to my mind, this should become a necessary qualification up to a certain point, as reports are frequently needed at once, and certainly she has every opportunity of acquiring this practised eye.

It is interesting to note that for the Cambridge Diploma *x*-ray and medical electricity are treated as one profession. The exceeding interest and breadth of this profession, with its infinite possibilities in all directions, can only be appreciated by those who have entered it—and entered it heart and soul. Hitherto the training and subsequent position of the radiographer have not been all that they should be; but this is perhaps only natural considering the comparative youth of the art. The Cambridge Diploma will soon fix the standard for the radiologist, and the same must be done for the radiographer. The incorporation of the Society of Radiographers is a step towards this end. Its objects are to promote the science and regulate the practice of radiography and to consider and discuss all subjects affecting it, to hold classes and examinations, and to award diplomas and certificates, such diplomas and certificates being issued by the authority of the Society only, and not by Government authority. The all-important step is that of training the pupils, and giving to the already trained every chance of reaping what they have sown by suitable examinations and practical tests. Let everyone's knowledge and capability have a fair trial, so that the good may be admitted, the bad cast out, and the specially good win honours. All this will come to pass provided that the present-day radiographers insist upon a high standard of efficiency for their work, and the students set themselves to reach that standard, which, when once attained, will entitle them to better conditions of remuneration and status than have hitherto in general existed.

---

Owing to the strike of the commercial printers which has extended all over Canada since May 1, our contemporary, *The Canadian Nurse*, has not been issued since April. It is hoped to bring out the July number, and possibly subscription dates may be advanced two months so that no hardship is inflicted on subscribers.

* The first article appeared June 11.

## Figures and Tables

**Figure f1:**